# Supplementary material for: The dynamics of functional brain network segregation in feedback-driven learning
Source: Commun Biol. 2024 May 6;7:531. doi: 10.1038/s42003-024-06210-9 (PMC11074323; doi:10.1038/s42003-024-06210-9)
Supplement: Supplementary file 2 — Supplementary Information [file 42003_2024_6210_MOESM2_ESM.pdf]

# Supplementary Information

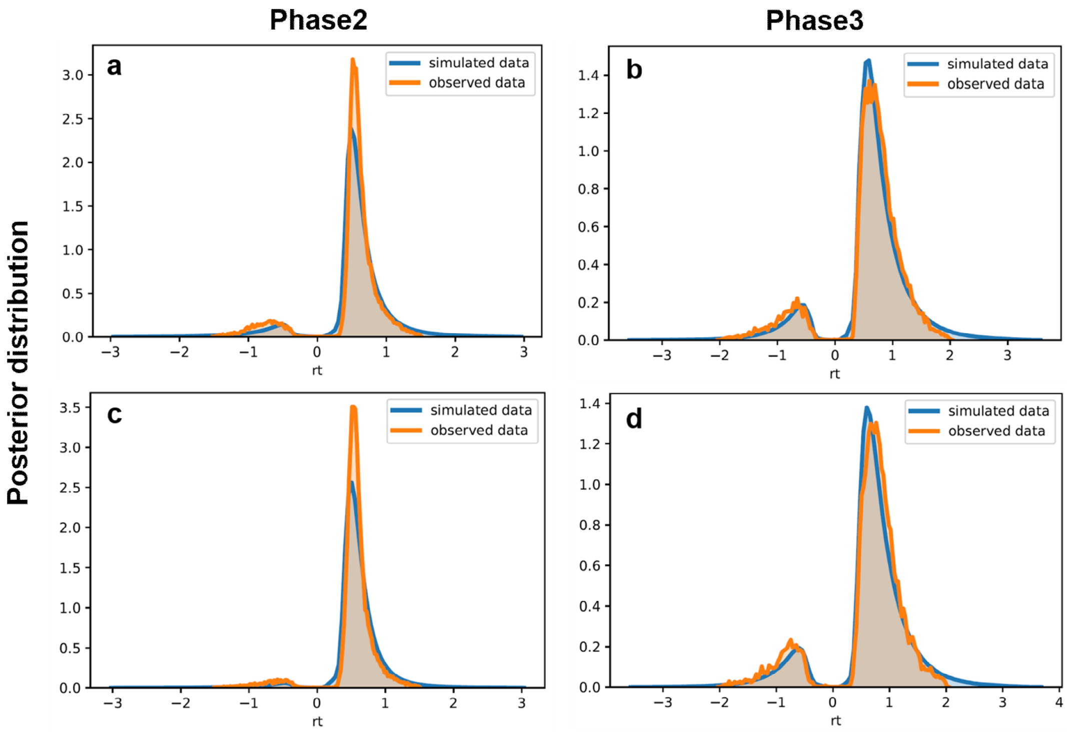


**Supplementary Figure 1.** Posterior predictive data distributions. Histogram shows normalized observed and simulated reaction time distribution (positive vs. negative results on the x-axis correspond to correct vs. incorrect responses) in phase 2 of study 1 (a) and study 2 (c) as well as in phase 3 of study 1 (b) and study 2 (d).

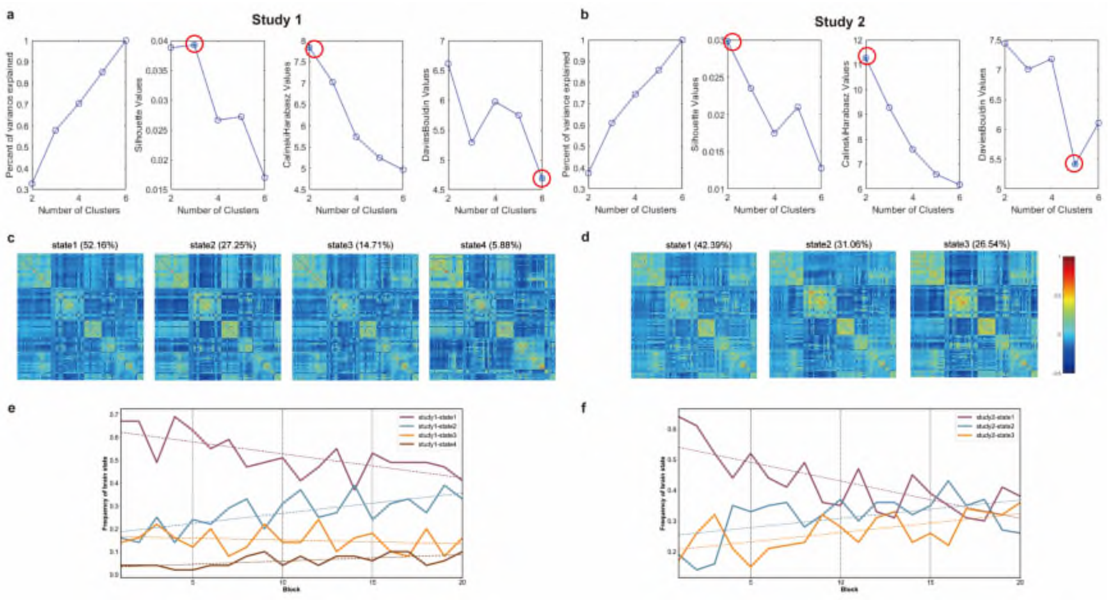

**Supplementary Figure 2**. The standard elbow results and three different methods determining the optimal number of clusters in k-means clustering for study 1 (a) and study 2 (b). The optimal number from the three standard criteria (Sihouette, CalinskiHarabasz, and DaviesBouldin) from the dynamic BC toolbox was 3, 2, and 6 in study 1 and 2, 2, and 5 in study 2 as highlighted by red circles. Following the guidelines of the dynamic BC toolbox, the optimal k-value is given by the mean value of these three criteria. Therefore, the best k values are 4 in study 1 and 3 in study 2. Please note that the results derived from the DaviesBouldin strongly deviates from the other two methods. Using the median instead would result in optimal k values of 3 and 2, respectively. Cluster centroids derived from k-means analysis in study 1 (c) and study 2 (d) are shown as connectivity matrices. The fourth and third brain states in study 1 are rather 'steady' with little meaningful change across learning. Moreover, the third and fourth states are only weakly present in all time windows (5.88% for state 4 and 14.71 % for state 3) compared to the other two brain states (52.16% for state 1 and 27.25 % for state 2). In study 2, the third state strongly resembled the second state and was therefore considered to be rather redundant. The evolution of different brain states is depicted in terms of frequency of occurrence of each brain state across consecutive learning blocks in study 1 (e) and study 2 (f).

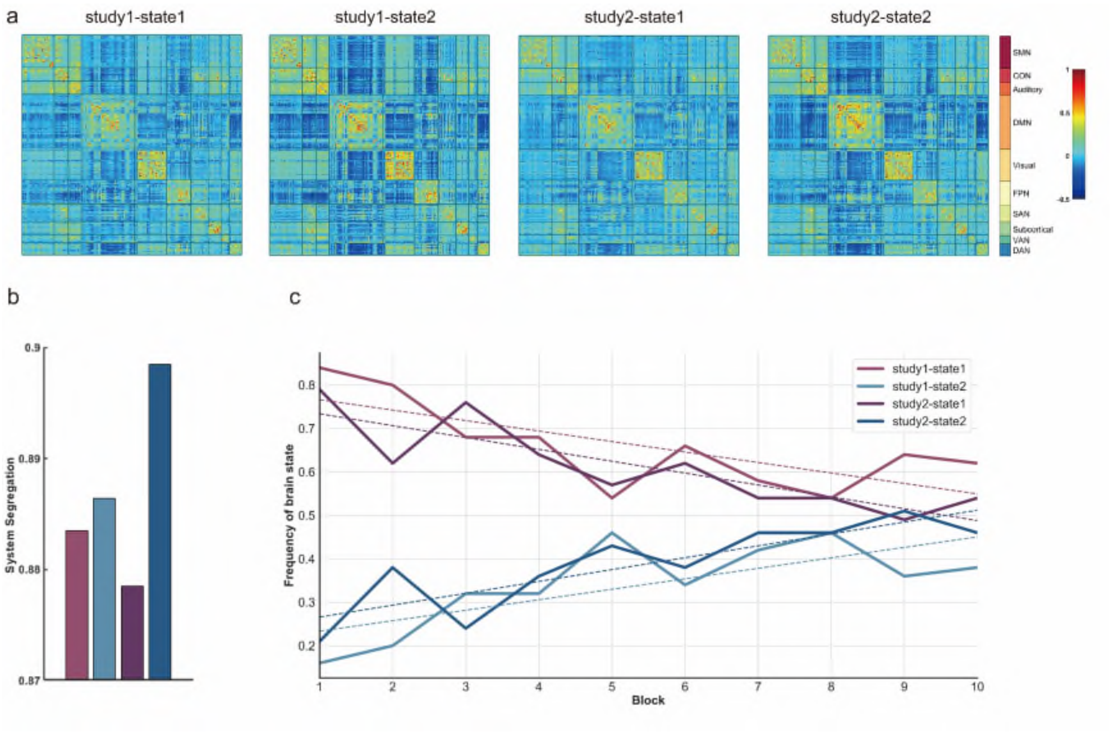


**Supplementary Figure 3.** Dynamics of brain states during learning (10 windows). (a) Cluster centroids derived from k-means analysis in study 1 and study 2 are shown as connectivity matrices; (b) Whole-brain system segregation computed from cluster centroid matrices; (c) Changes in frequency of the occurrence of integrated and segregated brain states during learning in study 1 and study 2.

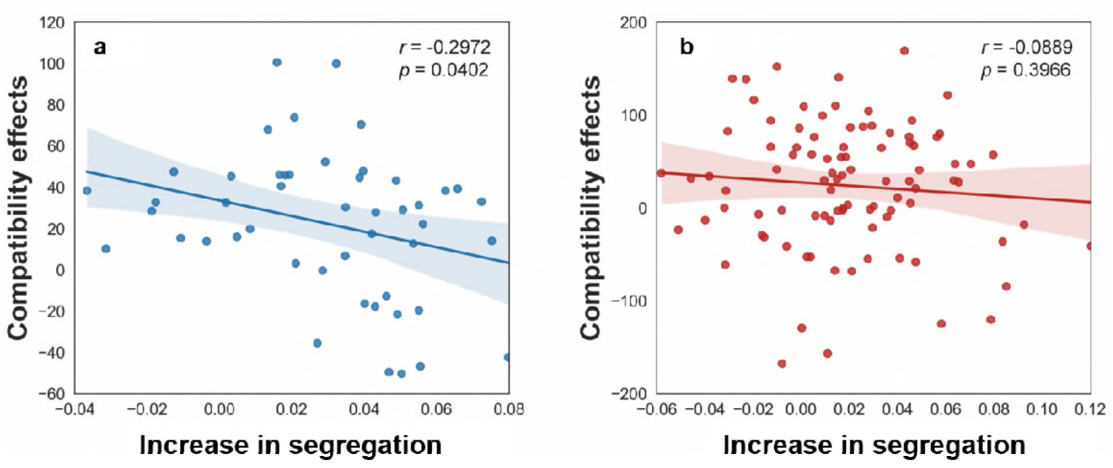


**Supplementary Figure 4.** Correlation between the size of the compatibility effect (habit strength) in phase 3 and the slope of modularity-Q value in study 1 (a) and study 2 (b). Here, different from the

primary analysis, the compatibility effect was computed for each subject as the response time difference between the incompatible and compatible conditions. Although, there was a significant effect in study 1, this was not replicated in study 2. Hence, together with the primary analysis results (compatibility effect based on drift rate), we concluded that there is no reliable relationship between habit strength and slope of modularity-Q value.

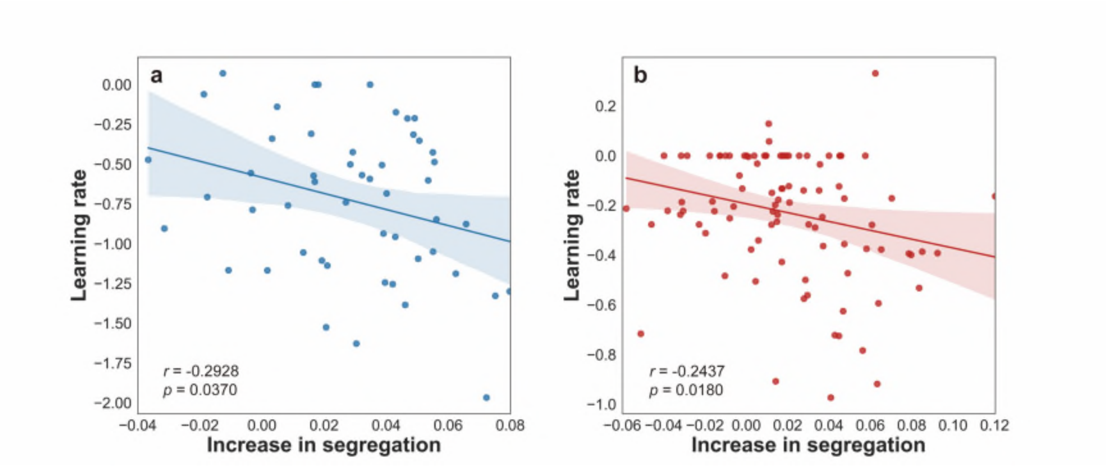


**Supplementary Figure 5.** Correlation between the learning rate in phase 2 and the slope of modularity-Q value in study 1 (a) and study 2 (b). Here, different from the primary analysis, learning rate was based on performance accuracy averaged across different stimuli as a function of the logarithm of the number of repetitions. As in the primary analysis, to obtain a single parameter per subject, we again used the negative exponent coefficient of the power function which was fitted to the individual accuracy-based learning curves.

**Supplementary Table 1 One sample t-tests on the slope of Participation Coefficient and Module Degree Z-score of each functional brain network**

| | ***study 1*** | ***study 2*** |
|---|---|---|

| Network | *Participation Coefficient* | | *Module Degree Z-score* | | *Participation Coefficient* | | *Module Degree Z-score* | |
|---|---|---|---|---|---|---|---|---|
| | ***t*** | ***p*** | ***t*** | ***p*** | ***t*** | ***p*** | ***t*** | ***p*** |
| Auditory | **-3.1680** | **< .001** | 1.6453 | 0.1063 | -1.5486 | 0.1249 | -0.8867 | 0.3775 |
| CON | -2.8710 | 0.0060 | 1.2440 | 0.2194 | -1.7176 | 0.0892 | -0.3859 | 0.7004 |
| DAN | -1.9868 | 0.0525 | 1.1757 | 0.2454 | -2.3983 | 0.0185 | 0.3139 | 0.7543 |
| DMN | **-5.6887** | **< .0001** | **3.1267** | **< .001** | **-4.9946** | **< .0001** | **4.3735** | **< .0001** |
| FPN | -0.0348 | 0.9724 | **-5.8506** | **< .0001** | -0.7075 | 0.4810 | **-3.0984** | **< .001** |
| SMN | -1.6691 | 0.1015 | -1.2180 | 0.2290 | -0.9964 | 0.3217 | -0.5364 | 0.5930 |
| SN | **-3.0978** | **< .001** | 0.0700 | 0.9445 | **-2.9778** | **< .001** | 0.5301 | 0.5973 |
| SUB | -2.3062 | 0.0254 | -0.7923 | 0.4320 | -2.1580 | 0.0335 | -1.3836 | 0.1698 |
| VAN | **-3.0857** | **< .001** | 0.3947 | 0.6948 | -2.2028 | 0.0301 | -2.2204 | 0.0288 |
| Visual | -2.1194 | 0.0391 | 1.6536 | 0.1046 | -1.1708 | 0.2447 | 0.2322 | 0.8169 |

The functional brain network which passed the FWE-correction were highlighted in bold font. CON, Cingulo-Opercular network; DAN, Dorsal attention network; DMN, Default-Mode Network; FPN, Fronto-Parietal network; SMN, Sensorimotor network; SN, Salience network; SUB, Subcortical network; VAN, Ventral attention network.
