# Supplementary material for: The dynamics of functional brain network segregation in feedback-driven learning
Source: Commun Biol. 2024 May 6;7:531. doi: 10.1038/s42003-024-06210-9 (PMC11074323; doi:10.1038/s42003-024-06210-9)
Supplement: Supplementary file 5 — Reporting Summary [file 42003_2024_6210_MOESM5_ESM.pdf]

nature portfolio

Corresponding author(s): xiaoyu wang COMMSBIO-23-4251A

Last updated by author(s): Apr 3, 2024

Reporting Summary

Nature Portfolio wishes to improve the reproducibility of the work that we publish. This form provides structure for consistency and transparency in reporting. For further information on Nature Portfolio policies, see our Editorial Policies and the Editorial Policy Checklist.

Statistics

For all statistical analyses, confirm that the following items are present in the figure legend, table legend, main text, or Methods section.

n/a	Confirmed	
☐	☒	The exact sample size (*n*) for each experimental group/condition, given as a discrete number and unit of measurement
☐	☒	A statement on whether measurements were taken from distinct samples or whether the same sample was measured repeatedly
☐	☒	The statistical test(s) used AND whether they are one- or two-sided<br>*Only common tests should be described solely by name; describe more complex techniques in the Methods section.*
☐	☒	A description of all covariates tested
☐	☒	A description of any assumptions or corrections, such as tests of normality and adjustment for multiple comparisons
☐	☒	A full description of the statistical parameters including central tendency (e.g. means) or other basic estimates (e.g. regression coefficient) AND variation (e.g. standard deviation) or associated estimates of uncertainty (e.g. confidence intervals)
☐	☒	For null hypothesis testing, the test statistic (e.g. *F*, *t*, *r*) with confidence intervals, effect sizes, degrees of freedom and *P* value noted<br>*Give P values as exact values whenever suitable.*
☐	☒	For Bayesian analysis, information on the choice of priors and Markov chain Monte Carlo settings
☒	☐	For hierarchical and complex designs, identification of the appropriate level for tests and full reporting of outcomes
☒	☐	Estimates of effect sizes (e.g. Cohen's *d*, Pearson's *r*), indicating how they were calculated

Our web collection on statistics for biologists contains articles on many of the points above.

Software and code

Policy information about availability of computer code

Data collection	The whole experiment is controlled by E-Prime 2.0; MRI data were acquired on a Siemens 3T whole body Trio System (Erlangen, Germany) with a 32 channel head coil.
Data analysis	Behavior data were analyzed with SPSS 20 and drift diffusion modeling were analyzed with Hierarchical Drift Diffusion Modeling (HDDM). The fMRI data were analyzed with SPM12 and Brain Connectivity Toolbox (BCT) based on MATLAB R2018b.

For manuscripts utilizing custom algorithms or software that are central to the research but not yet described in published literature, software must be made available to editors and reviewers. We strongly encourage code deposition in a community repository (e.g. GitHub). See the Nature Portfolio guidelines for submitting code & software for further information.

Data

Policy information about availability of data

All manuscripts must include a data availability statement. This statement should provide the following information, where applicable:

- Accession codes, unique identifiers, or web links for publicly available datasets
- A description of any restrictions on data availability
- For clinical datasets or third party data, please ensure that the statement adheres to our policy

The data that support the findings of this study are available on request from the corresponding author.

Research involving human participants, their data, or biological material

Policy information about studies with human participants or human data. See also policy information about sex, gender (identity/presentation), and sexual orientation and race, ethnicity and racism.

Reporting on sex and gender	N/A. The results apply to both biological sexes and any gender.
Reporting on race, ethnicity, or other socially relevant groupings	N/A.
Population characteristics	See above.
Recruitment	Participants were enrolled through the use of social media.
Ethics oversight	Technische Universität Dresden

Note that full information on the approval of the study protocol must also be provided in the manuscript.

Field-specific reporting

Please select the one below that is the best fit for your research. If you are not sure, read the appropriate sections before making your selection.

☐ Life sciences ☒ Behavioural & social sciences ☐ Ecological, evolutionary & environmental sciences

For a reference copy of the document with all sections, see nature.com/documents/nr-reporting-summary-flat.pdf

Behavioural & social sciences study design

All studies must disclose on these points even when the disclosure is negative.

Study description	Capitalizing on two separate fMRI studies using similar but not identical experimental designs, we set out to characterize performance improvement during feedback-driven stimulus-response (S-R) learning by learning rate as well as habit strength and tested whether and how these two distinct behavioral measures were associated with a functional brain state transition from a more integrated to a more segregated brain state across learning. We demonstrated for both studies that a higher learning rate was associated with a more rapid brain network segregation. By contrast, S-R habit strength was not reliably related to changes in brain network segregation.
Research sample	Fifty subjects (28 females, 22 males; mean age: 23 years, range 19-31 years) were included in study 1 and ninety-four subjects (all females, mean age: 19 years, range 12-30 years) were included in study 2. All subjects were right-handed, neurologically healthy, had normal or corrected vision, and normal color vision.
Sampling strategy	The present study employed the G-power software to determine the optimal design and sample size to increase the generalizability of the findings and enhance the study's reliability and validity. The sample size for this study is consistent with similar studies.
Data collection	fMRI data collection.
Timing	2016-2020
Data exclusions	three subjects from study 1 and two subjects from study 2 were excluded due to excessive head movement.
Non-participation	N/A.
Randomization	This is within-design study where participants were not allocated to experimental groups.

Reporting for specific materials, systems and methods

We require information from authors about some types of materials, experimental systems and methods used in many studies. Here, indicate whether each material, system or method listed is relevant to your study. If you are not sure if a list item applies to your research, read the appropriate section before selecting a response.

Materials & experimental systems

n/a	Involved in the study
☒	☐ Antibodies
☒	☐ Eukaryotic cell lines
☒	☐ Palaeontology and archaeology
☒	☐ Animals and other organisms
☒	☐ Clinical data
☒	☐ Dual use research of concern
☒	☐ Plants

Methods

n/a	Involved in the study
☒	☐ ChIP-seq
☒	☐ Flow cytometry
☐	☒ MRI-based neuroimaging

Plants

Seed stocks

Report on the source of all seed stocks or other plant material used. If applicable, state the seed stock centre and catalogue number. If plant specimens were collected from the field, describe the collection location, date and sampling procedures.

Novel plant genotypes

Describe the methods by which all novel plant genotypes were produced. This includes those generated by transgenic approaches, gene editing, chemical/radiation-based mutagenesis and hybridization. For transgenic lines, describe the transformation method, the number of independent lines analyzed and the generation upon which experiments were performed. For gene-edited lines, describe the editor used, the endogenous sequence targeted for editing, the targeting guide RNA sequence (if applicable) and how the editor was applied.

Authentication

Describe any authentication procedures for each seed stock used or novel genotype generated. Describe any experiments used to assess the effect of a mutation and, where applicable, how potential secondary effects (e.g. second site T-DNA insertions, mosiacism, off-target gene editing) were examined.

Magnetic resonance imaging

Experimental design

Design type

task fmri with event-related design.

Design specifications

The experimental paradigm consisted of three consecutive phases. Phase 1 comprised 240 trials; Trials in phase 2 were clustered into seven task blocks with 112 trials each (14 per stimulus). Hence, the whole phase 2 consisted of 784 trials (98 per stimulus); Phase 3 had 384 trials.

Behavioral performance measures

Response time and accuracy were collected.

Acquisition

Imaging type(s)

Structural and functional MRI data were collected.

Field strength

3 Tesla

Sequence & imaging parameters

Structural images were acquired using a T1-weighted sequence (TR = 1900 ms, TE = 2.26 ms, T1 = 900 ms, flip = 9°) with a resolution of 1 mm × 1 mm × 1 mm. Functional images were acquired using a gradient echo planar sequence (TR = 2000 ms, TE = 30 ms, flip angle = 80° in study 1; TR = 2070 ms, TE = 25 ms, flip angle = 80° in study 2). Each volume contained 32 slices (4 mm, 20% gap) that were measured in ascending order in study 1 whereas 36 slices (3.2 mm, 20% gap) were measured in descending order in study 2.

Area of acquisition

whole brain

Diffusion MRI ☐ Used ☒ Not used

Preprocessing

Preprocessing software

Data preprocessing was performed with SPM12 running in Matlab 9.5. The same processing steps were applied to study 1 and study 2 which included the following steps: discarding the initial 3 volumes; slice timing correction; motion correction; co-registering the T1-weighted images to the mean functional images and segmented it into gray matter (GM), white matter (WM) and cerebrospinal fluid (CSF); spatial normalization (3 mm resolution); nuisance regression which included the original six motion parameters, average signals in WM, CSF masks and their expansions (the first-order temporal derivative, as well as their squares and squared derivatives) as well as the whole brain signal; spatial smoothing (6 mm FWHM).

Normalization

standard SPM Normalize write option with images for warping and deformation Field.

Normalization template

Montreal neurological institute (MNI) reference space.

Noise and artifact removal

Nuisance regression which included the original six motion parameters, average signals in WM, CSF masks and their expansions (the first-order temporal derivative, as well as their squares and squared derivatives) as well as the whole brain signal. Subjects with spike events, diagnosed as the frame-wise displacement over 0.2 mm, in more than 20% of the fMRI data samples were excluded from further analysis. Three subjects in study 1 and two subjects in study 2 were excluded. In addition, we also tested whether head movement artifacts were responsible for individual differences in brain state dynamics. No correlation was observed between the measures of head movement artifacts (mean relative and max absolute head displacement) and the slope of modularity-Q value from the modularity analysis mentioned below for both study 1 and study 2 (all p-value > 0.05).

Volume censoring

N/A.

Statistical modeling & inference

Model type and settings

State-based functional connectivity statistics. In order to improve the test-retest reliability and to reduce spurious correlations between different brain regions in task-based functional connectivity analyses, the average task-related activity was regressed out. To this end, we performed the single-subject GLM analysis to obtain the residual time series for each subject which were then used for further functional connectivity computation. Learning trials were assigned to the correct approach or avoidance and error trials (irrespective of approach and avoidance) in study 1 and correct avoidance and error trials in study 2 separately. To appropriately capture BOLD activation, we used Fourier basis set regressors including 14 different sine-wave regressors spanning 30 s which were time-locked to the onset of the learning trials. After that, only for study 1 (no voluntary break in study 2), breaks between task blocks were also included as regressors with an additional GLM, the break-related regressors were based on the standard hemodynamic response function of SPM12 and convolved with the duration of breaks which varied considerably. With each subject-specific GLM, the high-pass filter was set to a cutoff of 128 s in SPM12, and estimated with ordinary least squares (that is, AR (1) off).

Effect(s) tested

1) The dynamic functional connectivity across the refined 227 Power nodes associated with 10 different functional networks was then calculated using 20 exponentially tapered sliding windows without overlap in study 1 and half-window overlapping in study 2. 2) A k-means clustering algorithm was applied to all windowed connectivity matrices (subjects × windows) using city block distance as the similarity measure. 3) the system segregation of each resulting FC cluster centroid was computed to examine the relative strength of within-network connectivity compared to between-network connectivity of the two different brain states derived from k-means. 4) the Louvain modularity algorithm from the Brain Connectivity Toolbox (BCT) was applied to investigate the optimal modular structure within the functional connectivity matrix by optimizing a quality function Q that maximizes within-module connectivity and minimizes between-module connectivity. 5) The slope of the modularity Q-value curve across 20 consecutive windows was used as a measure of the network segregation dynamics for each subject. Pearson correlation between the dynamics of network segregation and both the learning rate in phase 2 and habit strength in phase 3 were calculated to examine the relationship between fluctuations in network topology and behavioral performance in goal-habit transition. 6) the participation coefficient (PC) and Module-degree Z (MDZ) for each individual functional brain network node were also computed separately for each time window.

Specify type of analysis: ☐ Whole brain ☒ ROI-based ☐ Both

Anatomical location(s)

Power 227 nodes (Cole, M. W. et al. Multi-task connectivity reveals flexible hubs for adaptive task control. Nat Neurosci 16, 1348-1355, doi:10.1038/nn.3470 (2013))

Statistic type for inference

(See Eklund et al. 2016)

First, we tracked changes in brain network segregation and integration during goal-habit transition. The transition between the two different brain states was quantified using dynamic sliding window functional connectivity analysis. For each time window this method estimates correlations between multiple brain regions over successive time points which are then clustered into sets of recurring patterns, so-called dynamic connectivity states (DCS). Second, we investigated the relationship between the individual transition rate of the two different brain states (i.e., integrated vs. segregated states) as well as individual functional brain networks and individual task performance (learning rate and habit strength) as quantified via hierarchical drift diffusion modeling (HDDM).

Correction

FWE-correction for individual functional brain networks

Models & analysis

n/a	Involved in the study
☐	☒ Functional and/or effective connectivity
☐	☒ Graph analysis
☒	☐ Multivariate modeling or predictive analysis

Functional and/or effective connectivity

The residual time course were used to computed functional connectivity (as mentioned in Model type and settings part). The residual signals across all voxels within each ROI were averaged and Fisher z-transformed for the functional connectivity analysis. The dynamic functional connectivity across the refined 227 Power nodes associated with 10 different functional networks was then calculated using 20 exponentially tapered sliding windows without overlap in study 1 and half-window overlapping in study 2. We chose overlapping windows in study 2 to obtain the same number of windows as in study 1 considering that time series were only half as long as in study 1. To make sure that our results are independent of the arbitrary choice regarding number, overlap, and length of the sliding window, we repeated the analyses using 10 tapered sliding windows without overlap. This supplementary analysis showed similar results as the original analysis in both data sets (Supplementary Figure S2).
Tapering provides better suppression of spurious correlations and may reduce sensitivity to outliers and was

defined by the weight vector $w_t = w_0 e^{(t-T)/\theta}$, $t = 1, \ldots, T$, and $w_0 = (1 - e^{-1/\theta}) / (1 - e^{-T/\theta})$. The parameter t is the tth time point within the sliding window, N is the sliding window length, and the exponent θ controls the influence from distant time points. θ was set to a third of the window length, consistent with previous studies. We then constructed a functional connectivity matrix by computing the weighted Pearson correlation between time-series of any two nodes x_t and y_t for each time window and finally Fisher z-transformed the resulting weighted Pearson correlation matrix for the subsequent analyses.

Graph analysis

The Louvain modularity algorithm from the Brain Connectivity Toolbox (BCT) was applied to investigate the optimal modular structure within the functional connectivity matrix by optimizing a quality function Q that maximizes within-module connectivity and minimizes between-module connectivity. Higher modularity values (Q) therefore indicate stronger separation of networks. For each time window, the community assignment for each node, within which each node was assigned to its own community, was assessed 500 times and a consensus partition was identified using a fine-tuning algorithm from the BCT (http://www.brain-connectivity-toolbox.net/), which afforded an estimate of both the modularity values and community assignment for further analysis. All graph theoretical measures were calculated on weighted and signed connectivity matrices to avoid use of arbitrary thresholds, overcoming limitations of information loss. The γ parameter was set to 1.
